# Circulating Serum Exosomal Long Non-Coding RNAs FOXD2-AS1, NRIR, and XLOC_009459 as Diagnostic Biomarkers for Colorectal Cancer

**DOI:** 10.3389/fonc.2021.618967

**Published:** 2021-03-12

**Authors:** Miao Yu, Xing-guo Song, Ya-jing Zhao, Xiao-han Dong, Li-min Niu, Zhi-jun Zhang, Xiao-ling Shang, You-yong Tang, Xian-rang Song, Li Xie

**Affiliations:** ^1^ Shandong Provincial Third Hospital, Cheeloo College of Medicine, Shandong University, Jinan, China; ^2^ Department of Clinical Laboratory, Shandong Cancer Hospital and Institute, Shandong First Medical University and Shandong Academy of Medical Sciences, Jinan, China; ^3^ Shandong Provincial Key Laboratory of Radiation Oncology, Shandong Cancer Hospital and Institute, Shandong First Medical University and Shandong Academy of Medical Sciences, Jinan, China; ^4^ Department of Clinical Laboratory, Jinan Qilu Medical Inspection Co., Ltd., Jinan, China; ^5^ Department of Clinical Laboratory, Tai’an City Central Hospital, Tai’an, China

**Keywords:** exosomes, lncRNA, CRC, diagnosis, biomarker

## Abstract

**Background:**

Exosomes derived from cancer cells encapsulate various kinds of tumor-specific molecules and thus can interact with adjacent or distant cells to mediate information exchange. Long non-coding RNAs (lncRNAs) in exosomes have the potential as diagnostic and prognostic biomarkers in different types of cancers. The current study was aimed to identify circulating exosomal lncRNAs for the diagnosis of colorectal cancer (CRC).

**Methods:**

Exosomes were isolated from the serum by ultracentrifugation and verified by transmission electron microscope (TEM), qNano, and immunoblotting. Exosomal lncRNAs FOXD2-AS1, NRIR, and XLOC_009459 were selected by lncRNA microarray and validated by qPCR in 203 CRC patients and 201 healthy donors. The receiver operating characteristic curve (ROC) was used to assess the diagnostic efficiency of serum exosomal lncRNAs.

**Results:**

Exosomal FOXD2-AS1, NRIR, and XLOC_009459 (TCONS_00020073) levels were significantly upregulated in 203 CRC patients and 80 early-stage CRC patients compared to 201 healthy donors, possessing the area under the curve (AUC) of 0.728, 0.660, and 0.682 for CRC, as well as 0.743, 0.660, and 0.689 for early-stage CRC, respectively. Notably, their combination demonstrated the markedly elevated AUC of 0.736 for CRC and 0.758 for early-stage CRC, indicating their potential as diagnostic biomarkers for CRC.

**Conclusions:**

Our data suggested that exosomal lncRNAs FOXD2-AS1, NRIR, and XLOC_009459 act as the promising biomarkers for the diagnostics of CRC and early-stage CRC.

## Introduction

Colorectal cancer (CRC) remains one of the most common cancers worldwide, ranking third in men and second in women overall (1, 2). Early detection of CRC provides the best chances to prevent deaths from CRC, since the 5-year survival rate is 90% after surgical removal of localized tumor when diagnosed early, but dramatically decreases to 10% once it has metastasized (2, 3). Therefore, it is extremely important to find new non-invasive biomarkers with high sensitivity and specificity to improve different aspects of CRC management, just as fluid biopsy based on blood contents including cell-free DNA, circulating tumor cells, and exosomes.

Exosomes are extracellular vesicles with a diameter of approximately 30–100 nm (4). They can be secreted into the body fluid by many cells, including tumor and normal cells (5, 6). Exosomes derived from cancer cells encapsulate various kinds of tumor-specific molecules, such as nucleic acid and proteins (7, 8). Therefore, they can reflect biological characteristics of parental cells and interact with adjacent or distant cells to mediate information exchange (9–12). Recently, a number of studies have revealed that long noncoding RNAs (lncRNAs) are enriched and more stable in exosome, playing the crucial role in tumorigenesis and tumor development.

LncRNAs with a length of more than 200 base pairs do not encode proteins due to a lack of open reading frames. They can exert regulatory functions at different gene expression levels, including chromatin modification, transcription, and post-transcription. It can weaken the inhibitory or promotion effect of microRNAs on their downstream target genes by competitive binding, and thus affect their expression and function (13). Aberration expression of lncRNAs is observed in many cancers and plays a crucial role in tumorigenesis and tumor development. More importantly, lncRNAs seem to be measurable in body fluids, including the plasma, serum, and urine, empowering their potential as the non-invasive biomarkers for diagnostics of malignancies. For example, lncRNA XLOC_009167 can serve as a novel diagnostic biomarker for distinguishing lung cancer from benign lung disease and healthy controls (14); LINC00310, upregulated in breast tumors, is detected in the serum of breast cancer patients and serves as a promising diagnostic biomarker (15).

Accumulating evidence suggests that exosomal lncRNAs have an essential role in growth, metastasis, and invasion (16, 17). For example, a recent study has shown that exosomal lnc-Sox2ot acts as a ceRNA (competing endogenous RNA) by competitively binding to the miR-200 family to regulate the expression of Sox2 in pancreatic ductal adenocarcinoma (PDAC), promoting EMT, metastasis, and stem cell-like features of PDAC cells (18); Shujun et al. investigated the stability of exosomal lncRNAs. He revealed that expression levels of exosomal lncRNAs remained unchanged upon RNase A treatment and room temperature incubation test (19). Lin et al. confirmed that exosomal lncUEGC1 might be used as sensitive and stable circulating biomarkers for early-stage gastric cancer (20). Consequently, exosomal lncRNAs have potential as novel biomarkers for cancers (21–23).

In the present study, we aimed to identify circulating exosomal lncRNAs for CRC diagnosis. Exosomal lncRNAs FOXD2-AS1, NRIR, and XLOC_009459 were selected by lncRNA microarray and validated by qPCR in 203 CRC patients and 201 healthy donors. Our data demonstrated they were significantly upregulated in CRC and early-stage CRC with the favorable diagnostic efficiency, thus acting as the promising biomarkers for the diagnostics of CRC and early-stage CRC.

## Materials and Methods

### Patients and Healthy Donors

A total of 207 CRC patients, 203 healthy donors, and 20 patients with benign intestinal diseases (BIDs) admitted to Shandong Cancer Hospital and Institute between February 2018 and September 2019 were enrolled in the current study. All patients didn’t receive any anti-tumor treatment before sampling, or suffer from any other endocrine, immune, or metabolic diseases. Healthy donors did not present any disease. TNM classification was estimated according to the AJCC Cancer Staging Handbook of the American Joint Committee on Cancer 2010. This study was approved by Ethics Committee of Shandong Cancer Hospital affiliated to Shandong First Medical University and Shandong Academy of Medical Sciences. Informed consent was obtained from all individuals.

### LncRNA Sequence Data Sets and Analysis

Arraystar Human LncRNA Microarray V4.0 (KangCheng, Shanghai, China) was used for lncRNA microarray analysis. Total RNA was extracted from serum exosomes of 4 CRC patients and 2 healthy volunteers. RNA quantity and quality were measured by NanoDrop ND-1000 (Agilent Technology, California, USA), followed by Sample labeling using Arraystar RNA Flash Labeling Kit (Agilent Technology) as well as array hybridization using Agilent SureHyb (Agilent Technology). Agilent Gene GX v12.1 software was used to perform chip standardization and screen out differential lncRNAs.

### Exosomes Isolation

Exosomes were extracted from 1 mL human serum using ultracentrifugation as previously described (24, 25). In brief, the serum was centrifuged at 10,000×g for 30 min at 4°C to remove the cellular debris, followed by ultracentrifugation (Beckman Coulter, Brea, CA, USA) at 100,000×g for 2 h at 4°C, after which exosomal pellets were re-suspended in PBS for further analysis.

### Transmission Electron Microscopy (TEM)

The extracted exosomal samples were dissolved in 100 μL PBS, instantaneously centrifuged, after which 15 μL specimen was pipetted on copper wire for 1 min (copper tweezers were used to lightly clamp the copper mesh and to prevent it from breaking). The specimen was dried with filter paper and stained using 15 μL of 2% uranyl acetate for 1 minute at room temperature. The stained samples were dried and roasted under the lamp for 10 minutes. The morphology of isolated exosomes was visualized by transmission electron microscopy (Tecnai, Oregon State, USA).

### qNano

Isolated exosomes were diluted with PBS and thoroughly mixed. The exosomes’ size and particle concentration were analyzed by qNano (Izon Science Ltd, Christchurch, New Zealand). Particle concentration was standardized by calibration beads of 1.0 × 10^13^ particles/mL. Data were analyzed using Izon Control Suite v.3.3.2.2000 (Izon Science Ltd).

### Immunoblotting

Total exosomal and cellular proteins were separated using 12% SDS-PAGE and transferred onto a PVDF membrane (Millipore, Billerica, MA, USA). The membranes were blocked with 5% evaporated skimmed milk in TBST (50 mmol/L Tris-HCl, pH 7.5, 150mmol/L NaCl) containing 0.1% Tween-20 for 1 hour. Samples were then incubated overnight at 4°C with the appropriate primary antibodies, including antibodies against CD9, TSG101, and GM130 (Cell Signaling Technology, USA), followed by incubation with HRP-coupled secondary antibodies (Proteintech Group, China) for 1 hour at room temperature. Furthermore, the protein bands were visualized on photographic film using ECL blotting detection reagents (Bio-Rad, USA).

### RNAs Extraction and qRT-PCR

Total RNA was extracted from exosomes using Trizol reagent (Thermo Fisher Scientific, Carlsbad, CA, USA) according to the manufacturer’s protocol. The extracted RNA was reverse-transcribed into cDNA using the PrimeScript™ RT reagent Kit (Takara Bio, Kusatsu, Japan) according to the manufacturer’s instructions. qRT-PCR was performed using TB-Green Premix Ex Taq II Reagent (TaKaRa Bio) on an LC480 (Roche Diagnostics, Germany) according to the manufacturer’s instructions. ACTB was used as an internal control. Each sample was analyzed in duplicate. The relative expression levels of exosomal lncRNAs were normalized to ACTB using the ΔCT method (Ct^lncRNA^-Ct^ACTB^) as previously described (26). The primers (Biosune biotechnology, Shanghai) sequences used in this study are shown in **Table 1**.

**Table 1:** Sequence Information of the primers for qRT-PCR.

lncRNA	Forward primer	Reverse primer
FOXD2-AS1	GCCCAGAACAATTGGGAGGA	AAGAGAGGGAGAGACGACCC
NRIR	CCTTGGCAACTGCTCACGAT	AGGAGGTTAGAGGTGTCTGCT
XLOC_009459	GGTGGGATACGTGCCTCTTC	AGACAGTGCTGTGTGAGACG
ACTB	TTAGTTGCGTTACACCCTTTC	GCTGTCACCTTCACCGTTC

### Statistical Analysis

Graphpad prism version 6.0 (Graphpad, San Diego, CA, USA) and SPSS 22.0 software (IBM, Ehningen, Germany) were used for statistical analysis. The Shapiro-Wilk test was used to verify the distribution characteristics of the experimental data. Levene’s test was used to verify the homogeneity of variance. Unpaired t-test, ANOVA analysis, and paired t-test were used for data that followed normal distribution and homogeneity of variance; numerical results were expressed in mean and interquartile range. For data with non-normal distribution, the Mann-Whitney test was used between two groups; the Wilcoxon rank-test was used between two paired groups of data; the Kruskal-Wallis test was used among multiple groups; numerical results are expressed in median and interquartile range. The area under the curve (AUC) was calculated according to the receiver operating characteristic (ROC) curves to determine the efficiency of the diagnostic. P-value < 0.05 was considered as statistically significant difference, and all tests were set as double-tailed.

## Results

### Identification of the Differential Exosomal lncRNAs

**Figure 1:**
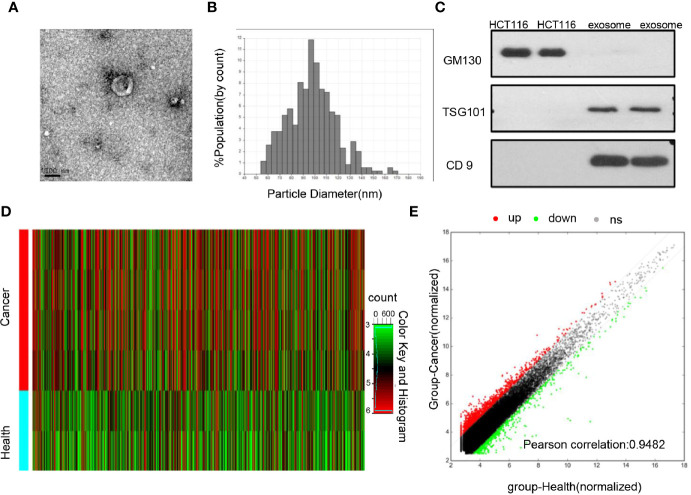
Identification of the differential exosomal lncRNAs **(A)** TEM images showed that exosomes were oval or bowl-shaped capsules without the nucleus. **(B)** QNano results suggested that the isolated exosome enriched from serum was about 50–150 nm in diameter. **(C)** Exosome markers TSG101 and CD9 were detected in the exosomes isolated from the serum; GM130, a negative marker of the exosome, was absent in our isolated exosome. **(D)** Hierarchical clustering analysis of differentially expressed lncRNAs among CRC patients and healthy donors using lncRNAs microarray. **(E)** Differential expression analysis between CRC patients and healthy donors were expressed with hybridization signals. The red dot represents upregulated lncRNAs expression in colorectal cancer, and the green dot represents downregulated lncRNAs expression.

First, the isolated exosomes were verified by TEM, qNano, and western blot analysis. As shown in **Figures 1A, B** they displayed special vesicles with double-layer membrane structure under transmission electron microscopy and measured 50–150 nm in diameter by qNano. The expressions of exosomal specific markers (CD9 and TSG101) were verified in the exosomes but were not detected in the cell lysis, whereas GM130 (the negative control) was only expressed in the cell lysis but not in exosomes (**Figure 1C**). In general, these results suggested that exosomes were successfully isolated by ultracentrifugation.

To explore the differential lncRNAs, exosomes isolated from 4 CRC primary patients and 2 healthy donors were subjected to microarray analysis. As show in **Figures 1D, E**, 1,131 upregulated and 808 downregulated lncRNAs with significantly different expression (fold change≥2.0 and P<0.05) were screened out. Among them, 16 lncRNAs with the most significance were selected including 7 upregulated genes (FOXD2-AS1, NRIR, XLOC_009459, MIAT, LINC001481, AP000892.4, FAM197Y9) and 9 downregulated genes (DICER1-AS1, LINC00570, MIRLET7BHG, LINC00885, HOTAIR, AC012456.3, DSCAM-AS1, MIR503HG, POC1B), then subjected to primer design and specificity verification, and 8 of them were excluded due to poor primer specificity; others were subjected to a cohort with 48 healthy people and 48 CRC patients, and 5 of them were excluded due to no difference. Finally, FOXD2-AS1 and NRIR and XLOC_009459 were identified and then subjected to a more expanded cohort for future validation (**Figure S1**).

### Stability of Exosomal lncRNAs FOXD2-AS1, NRIR, and XLOC_009459

**Figure 2:**
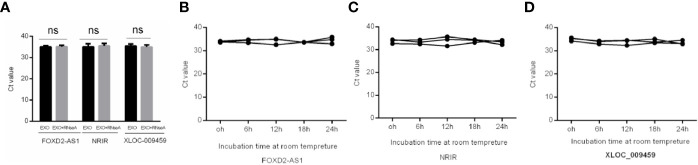
Stability of Exosomal lncRNAs FOXD2-AS1 and NRIR and XLOC_009459. **(A)** qRT-PCR analysis of the three lncRNAs in the exosomes or exosomes deleted with Rnase A **(B–D)** The expressions of the three serum exosomal lncRNAs when incubated at room temperature (ns, not significant).

To prove the stability of exosomal lncRNAs, the isolated exosomes were incubated with RNase A and stored at room temperature. As shown in **Figures 2A–D** the expression levels of FOXD2-AS1 and NRIR and XLOC_009459 confirmed that exosomes could protect its contained lncRNAs from degradation by RNase A, as well as room temperature incubation for 0, 6, 12, 18, and 24 hours, indicating the exosomal membrane could maintain the stability of lncRNAs.

### Exosomal lncRNAs FOXD2-AS1, NRIR, and XLOC_009459 Were Upregulated in CRC Patients

**Figure 3:**
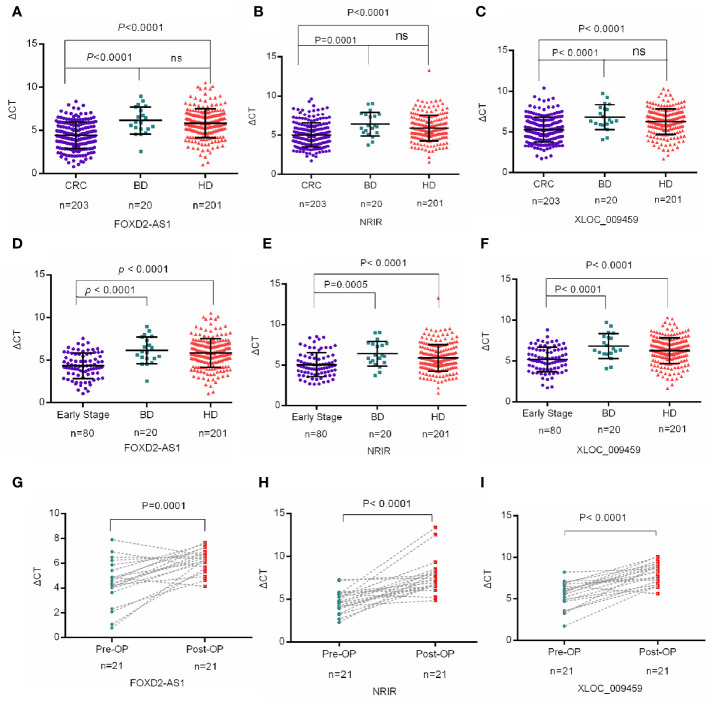
Exosomal lncRNAs FOXD2-AS1, NRIR, and XLOC_009459 were upregulated in CRC patients **(A–F)** Mann-Whitney U test indicated that the expression levels of serum exosomal FOXD2-AS1 and NRIR and XLOC_009459 were significantly upregulated in CRC patients (n=203) and early CRC patients (n=80) as compared to healthy donors (n=201) and benign intestinal diseases (BIDs) (n=20). The expression levels of FOXD2-AS1, NRIR, and XLOC_009459 showed no difference between benign intestinal diseases (BIDs) (n=20) and healthy donors (n=201). **(G–I)** The expression levels of exosomal FOXD2-AS1 and NRIR and XLOC_009459 were significantly different in 21 preoperative (Pre-OP) patients and paired postoperative (Post-OP) patients. ns, no significance.

We then analyzed the expression levels of serum exosomal FOXD2-AS1, NRIR, and XLOC_009459 in 203 CRC patients, 201 healthy donors, and 20 BIDs. As shown in **Figures 3A–C**, they were significantly upregulated in CRC patients compared with those in the healthy donors or in BIDs (*P*<0.0001, respectively). Nevertheless, no significant differences of their expression were observed between healthy donors and BIDs. The correlation between these three exosomal LncRNAs expression and clinicopathological characteristics was also evaluated. As shown in **Table 2**, exosomal XLOC_009459 was obviously associated with age but not with others.

**Table 2:** Correlation between exosomal FOXD2-AS1, NRIR and XLOC_009459 expression and clinicopathologic characteristics of CRC patients.

Factor		Cases	FOXD2-AS1	P value	NRIR	P value	XLOC_009459	P value
**Age(years)**	<61	99	4.6288(4.3403-4.9173)	0.088	4.9500(4.8695-5.4966)	0.133	5.5448(5.2163-5.8734)	**0.024**
	≥61	104	4.2642(3.9568-4.5716)		4.6600(4.5715-5.1481)		5.0606(4.7906-5.3305)	
**Gender**	Male	135	4.4459(4.1947-4.6970)	0.96	4.7700(4.7349-5.2492)	0.831	5.3349(5.0655-5.6043)	0.619
	Female	68	4.4344(4.0406-4.8283)		4.7800(4.6847-5.4509)		5.2210(4.8709-5.5711)	
**Drinking**	No	155	4.3970(4.1393-4.6548)	0.368	4.80(4.7799-5.2776)	0.751	5.2370(4.9914-5.4826)	0.146
	Yes	48	4.5873(4.2553-4.9193)		4.7250(4.5678-5.3939)		5.6015(5.1991-6.0038)	
**Diabetes**	No	181	4.3878(4.1594-4.6162)	0.146	4.9654(4.7398-5.1910)	0.131	5.2598(5.0309-5.4888)	0.327
	Yes	22	4.8882(4.3778-5.3986)		5.4459(4.8079-6.0839)		5.6005(5.0253-6.1756)	
**Tumor position**	Rectum	128	4.3080(4.0432-4.5727)	0.102	4.7650(4.6437-5.1555)	0.256	5.1481(4.8920-5.4043)	0.072
	Colon	55	4.6708(4.3202-5.0214)		4.85(4.8429-5.5942)		5.5504(5.1757-5.9251)	
	unknown	20						
**Histological**	poor	23	4.4848 (3.7876-5.1819)	0.51	5.1478(4.4490-5.8466)	0.471	5.1243(4.4066-5.8421)	0.258
	moderate	97	4.3439 (4.0420-4.6458)		4.8905(4.5974-5.1836)		5.1755(4.8937-5.4572)	
	well	4	5.21 (3.4654-6.9546)		5.6725(3.8635-7.4815)		6.3725(4.8988-7.8462)	
	unknown	79						
**Tumor size**	≥26	65	4.2760 (3.8519-4.7001)	0.244	4.69(4.4716-5.2228)	0.465	5.3538(4.9286-5.7791)	0.969
	<26	65	4.5923 (4.2574-4.9273)		4.95(4.7235-5.45689)		5.3429(4.9798-5.7061)	
	unknown	73						
**LN metastasis**	No	80	4.3544(4.0289-4.6798)	0.505	4.8450(4.0289-4.6798)	0.755	5.28(4.9068-5.5602)	0.924
	Yes	119	4.5008(4.2193-4.7822)		4.5008(4.2193-4.7822)		5.11(5.0605-5.6372)	
	unknown	4						
**Distant metastasis**	No	144	4.3793(4.1229-4.6357)	0.452	4.7950(4.7576-5.2586)	0.767	5.1050(4.9961-5.508)	0.783
	Yes	54	4.5607(4.1876-4.9339)		4.6(4.5228-5.3709)		5.2550(4.9908-5.8062)	
	unknown	5						
**TNM stage**	I	20	4.38(3.535-5.225)	0.716	5.1430(4.2657-6.0203)	0.999	5.4370(4.5109-6.3631)	0.696
	II	60	4.3158(3.9571-4.6746)		4.7250(4.6704-5.3726)		5.1167(4.7763-5.4570)	
	III	60	4.4875(4.0604-4.9146)		4.9885(4.5887-5.3883)		5.4007(4.9776-5.8237)	
	IV	54	4.6385(4.2595-5.0175)		4.6450(4.6112-5.5088)		5.2850(5.0364-5.8555)	
	unknown	9						

Bold value: P < 0.05.

The differential expression of exosomal FOXD2-AS1, NRIR, and XLOC_009459 was also analyzed between 80 early-stage CRC patients (TNM stage I+II) and 201 healthy donors or 20 BIDs. Consistently, all of them were significantly higher in early-stage CRC patients than those in healthy donors or BIDs (**Figures 3D–F**). Moreover, exosomal FOXD2-AS1 and NRIR and XLOC_009459 expression seemed irrelated with clinicopathological characteristics of 80 early-stage CRC patients including age, gender, drinking status, history of diabetes mellitus, tumor position, histological type, lymph node metastasis status, and TNM stage as shown in **Table 3**.

**Table 3:** Correlation between exosomal FOXD2-AS1 and NRIR and XLOC_009459 expression and clinicopathologic characteristics of early-stage CRC patients.

Factor		Cases	FOXD2-AS1	P value	NRIR	P value	XLOC_009459	P value
**Age(years)**	<60	38	4.2508(3.7927-4.7089)	0.282	4.64(4.5142-5.5532)	0.686	5.3234(4.7959-5.851)	0.477
	≥60	42	4.4052(3.9103-4.9002)		4.925(4.6236-5.513)		5.0821(4.6418-5.5225)	
**Gender**	Male	58	4.3184(3.9410-4.6959)	0.897	4.73(4.5894-5.3482)	0.484	5.2043(4.8116-5.5970)	0.942
	Female	22	4.3673(3.6287-5.1059)		4.98(4.5480-5.9939)		5.1768(4.4846-5.8691)	
**Drinking**	No	56	4.2995(3.8589-4.7400)	0.730	4.8(4.6826-5.4995)	0.741	5.1346(4.7337-5.5356)	0.576
	Yes	24	4.4075(3.9520-4.8630)		4.91(4.3522-5.5686)		5.3417(4.6924-5.9909)	
**Diabetes**	No	72	4.2738(3.9209-4.6266)	0.299	4.7250(4.6404-5.3435)	0.248	5.1715(4.8149-5.5282)	0.655
	Yes	8	4.8550(3.7015-6.0085)		5.3650(4.3895-6.7930)		5.4238(4.2261-6.6214)	
**Tumor position**	Rectum	42	4.3869(3.8502-4.9237)	0.871	4.9095(4.4319-5.3871)	0.590	5.1448(4.6107-5.6788)	0.764
	Colon	25	4.3216(3.7747-4.8685)		5.1188(4.4875-5.7501)		5.2668(4.6938-5.8398)	
	unknown	13						
**Histological**	poor	7	4.4984(4.0449-4.9520)	0.155	4.0943(2.9914-5.1972)	0.612	4.0943(2.9914-5.1972)	0.099
	moderate	51	4.99(1.9347-8.0453)		4.85(4.6849-5.5637)		5.2753(4.8366-5.7144)	
	well	3	4.99(1.9347-8.0453)		5.63(2.1808-9.0792)		6.23(3.5491-8.9109)	
	unknown	19						
**Tumor size**	<22.5	32	4.7403(4.2139-5.2668)	0.085	5.3841(4.7815-5.9867)	0.059	5.4719(4.8648-6.0789)	0.211
	≥22.5	33	4.0764(3.5116-4.6411)		4.6679(4.2022-5.1335)		4.9736(4.4453-5.5020)	
	unknown	15						

Moreover, we also searched and analyzed the expression of lncRNAs FOXD2-AS1 and NRIR in cancer and paracancerous tissues in the TCGA database. As shown in **Figure S2**, FOXD2-AS1 was upregulated in CRC tissues compared with the paracancerous, whereas NRIR had no difference in CRC and paracancerous tissues. However, the data of XLOC_009459 was absent in TCGA database since it was newly identified.

Finally, we studied the relationship between exosomal lncRNAs expression and tumor occupying *via* detecting the differences of these three lncRNAs expression pre- and post-operation of CRC. The expression levels of exosomal FOXD2-AS1 and NRIR and XLOC_009459 were significantly decreased after resection of the primary tumor (**Figures 3G–I**), suggesting that tumor burden may have an impact on the expression level of exosomal FOXD2-AS1 and NRIR and XLOC_009459.

### Exosomal lncRNAs FOXD2-AS1 and NRIR and XLOC_009459 as Diagnostics Biomarker for CRC

**Figure 4:**
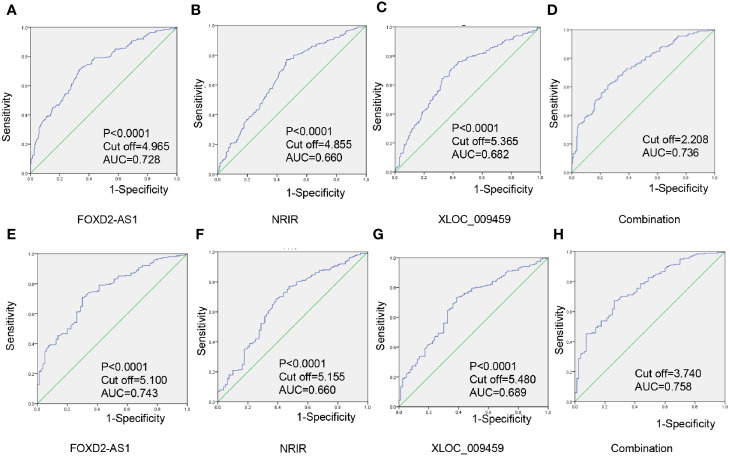
Exosomal lncRNAs FOXD2-AS1 and NRIR and XLOC_009459 as diagnostics biomarker for CRC **(A–D)** Individual and combined diagnosis value of serum exosomal FOXD2-AS1 and NRIR and XLOC_009459 in 203 CRC patients and 201 healthy donors. **(E–H)** An individual and combined diagnosis value of exosomal FOXD2-AS1 and NRIR and XLOC_009459 in 80 early-stage CRC patients and 201 healthy donors.

To evaluate diagnostic performance of exosomal FOXD2-AS1 and NRIR and XLOC_009459 for CRC, a ROC curve was calculated *via* comparing the 203 CRC patients and 201 healthy donors. The AUC of exosomal FOXD2-AS1 and NRIR and XLOC_009459 were 0.728 with 72.6% sensitivity and 62.3% specificity, 0.660 with 77.1% sensitivity and 69.2% specificity, and 0.682 with 76.1% sensitivity and 67.2% specificity, respectively (**Figures 4A–C**). We also calculated diagnostics accuracy of their combination, possessing the most powerful efficiency with an AUC of up to 0.736 with 61.2% sensitivity and 75% specificity (**Figure 4D**), indicating that these three exosomal lncRNAs potentially act as the non-invasive circulating biomarkers for CRC.

ROC curves were also employed to evaluate diagnostic performance for early-stage CRC. As shown in **Figures 4E–G**, the AUC of serum exosomal FOXD2-AS1 and NRIR and XLOC_009459 were 0.743 with 70.6% sensitivity and 59.4% specificity; 0.660 with 68.7% sensitivity and 70.1% specificity; and 0.689 with 73.1% sensitivity and 65.6% specificity. Besides, the diagnostics accuracy of their combination possessed an AUC of up to 0.758 with 67.2% sensitivity and 73.8% specificity (**Figure 4H**).

## Discussion

CRC is the third most common cancer and the third leading cause of cancer-related death worldwide, particularly in China (27). The discovery of new biomarkers for detecting CRC with high sensitivity and specificity might improve different aspects of CRC management. In the current study, our result demonstrated that exosomal FOXD2-AS1, NRIR, and XLOC_009459 were significantly upregulated in CRC and early CRC, thus revealing their potential as diagnostic biomarkers for CRC.

FOXD2-AS1, a 2527 bp lncRNA located on chromosome 1p33, is a promising candidate among all tumor-related lncRNAs (28). Related studies have shown that FOXD2-AS1 is upregulated in various malignancies, including gastric, lung, bladder, colorectal, etc., thus contributing to proliferation, migration, and invasion of tumor cells in various pathways (29), such as through their interaction with microRNA-185-5p (30); NRIR, also known as lncRNA-CMPK2, is a spliced, polyadenylated nuclear transcript induced by IFN in diverse cell types from human and mouse. It was upregulated in CRC tissues and positively correlated with clinical stages (31), as well as differentially expressed in hepatocellular carcinoma (HCC) patients with or without fibrosis and effectively predicted survival in HCC patients with or without fibrosis (32); XLOC_009459, a 607 bp lncRNA located on chromosome 11q13.1, was recently identified (33); nevertheless, its role and functions in cancer still remain undefined.

In the current study, several lines of evidence validated that exosomal lncRNAs FOXD2-AS1, NRIR, and XLOC_009459 acted as diagnostic biomarkers for CRC. First, three exosomal lncRNAs were significantly upregulated in CRC patients and early-stage CRC patients compared with healthy donors. Besides, they were significantly decreased after surgery, indicating they were closely correlated with tumor occupying. Second, these three exosomal LncRNAs possessed rather high diagnostic efficiency, not only for CRC but also for early CRC. Finally, this study also confirmed that exosomes could protect lncRNAs from degradation caused by RNase A and make lncRNAs stable in serum, thus suggesting that exosomal lncRNAs FOXD2-AS1, NRIR, and XLOC_009459 could serve as non-invasive tumor markers for CRC diagnosis and early-stage CRC patients.

However, there are several limitations in the present study that should be pointed out. First, our study included 207 CRC patients, 201 healthy donors, and 20 BIDs, thus the total sample size was small and short of statistically vigorous power. In addition, the roles of FOXD2-AS1, NRIR, and XLOC_009459 in the prognosis evaluation of CRC were not analyzed as we lacked long-term clinical follow-up data. Second, we failed to analyze the combined diagnosis efficacy of the three exosomal lncRNAs with the common tumor biomarker, such as CEA, because the relative information was missing in the healthy donor cohort. In the future, we plan to investigate the mechanisms and prognostic value of these three serum exosomal lncRNAs in CRC.

In summary, we found that serum exosomal FOXD2-AS1, NRIR, and XLOC_009459 were significantly upregulated in 203 CRC patients and 80 early-stage CRC patients compared to 201 healthy donors, showing higher upregulation with favorable diagnostic efficiency. Also, the relative lncRNAs expression was significantly decreased postoperatively as compared to the baseline levels determined before surgery, thus providing evidence that exosomal FOXD2-AS1, NRIR, and XLOC_009459 could be used as biomarkers for diagnosis of patients with CRC.

## Data Availability Statement

The raw data supporting the conclusions of this article will be made available by the authors, without undue reservation.

## Ethics Statement

This study was approved by the responsible committee for human experimentation of Shandong Cancer Hospital affiliated to Shandong First Medical University and Shandong Academy of Medical Sciences. Informed consent was obtained for all individuals.

## Author Contributions

LX designed experiments. MY carried out experiments. MY and X-GS wrote the manuscript and prepared figures. Y-JZ and X-HD provided the blood samples. L-MN, Z-JZ, X-LS, YT, and X-RS contributed to analysis the experimental data. All authors reviewed the manuscript. All authors contributed to the article and approved the submitted version.

## Funding

This work was supported by the National Natural Science Foundation of China (81773237), Shandong Provincial Natural Science Foundation (ZR2020LZL017), Shandong Province Medical and Health Technology Development Plan Project (2019WS455), and Jinan Science and Technology Program (201704080).

## Conflict of Interest

MY was employed by company Jinan Qilu Medical Inspection Co., Ltd.

The remaining authors declare that the research was conducted in the absence of any commercial or financial relationships that could be construed as a potential conflict of interest.

The reviewer YZ declared a shared affiliation, with no collaboration, with one of the authors MY, to the handling editor at the time of the review.

## Supplementary Material

Supplementary Figure 1 The flowchart for the exclusion criteria.

Supplementary Figure 2 The expression of lncRNAs FOXD2-AS1 **(A)** and NRIR **(B)** in cancer and paracancerous tissues in the TCGA database.

## Abbreviations

LncRNAs, Long non-coding RNAs; CRC, colorectal cancer; TEM, transmission electron microscope; qRT-PCR, Quantitative real-time polymerase chain reaction; BIDs, benign intestinal diseases; ΔCt, Comparative cycle threshold; ROC, Receiving operating characteristic; AUC, Area under the curve; CEA, Carcinoembryonic antigen.
