# Supplementary figures and images for: Circulating Serum Exosomal Long Non-Coding RNAs FOXD2-AS1, NRIR, and XLOC_009459 as Diagnostic Biomarkers for Colorectal Cancer
Source: Front Oncol. 2021 Mar 12;11:618967. doi: 10.3389/fonc.2021.618967 (PMC7996089; doi:10.3389/fonc.2021.618967)

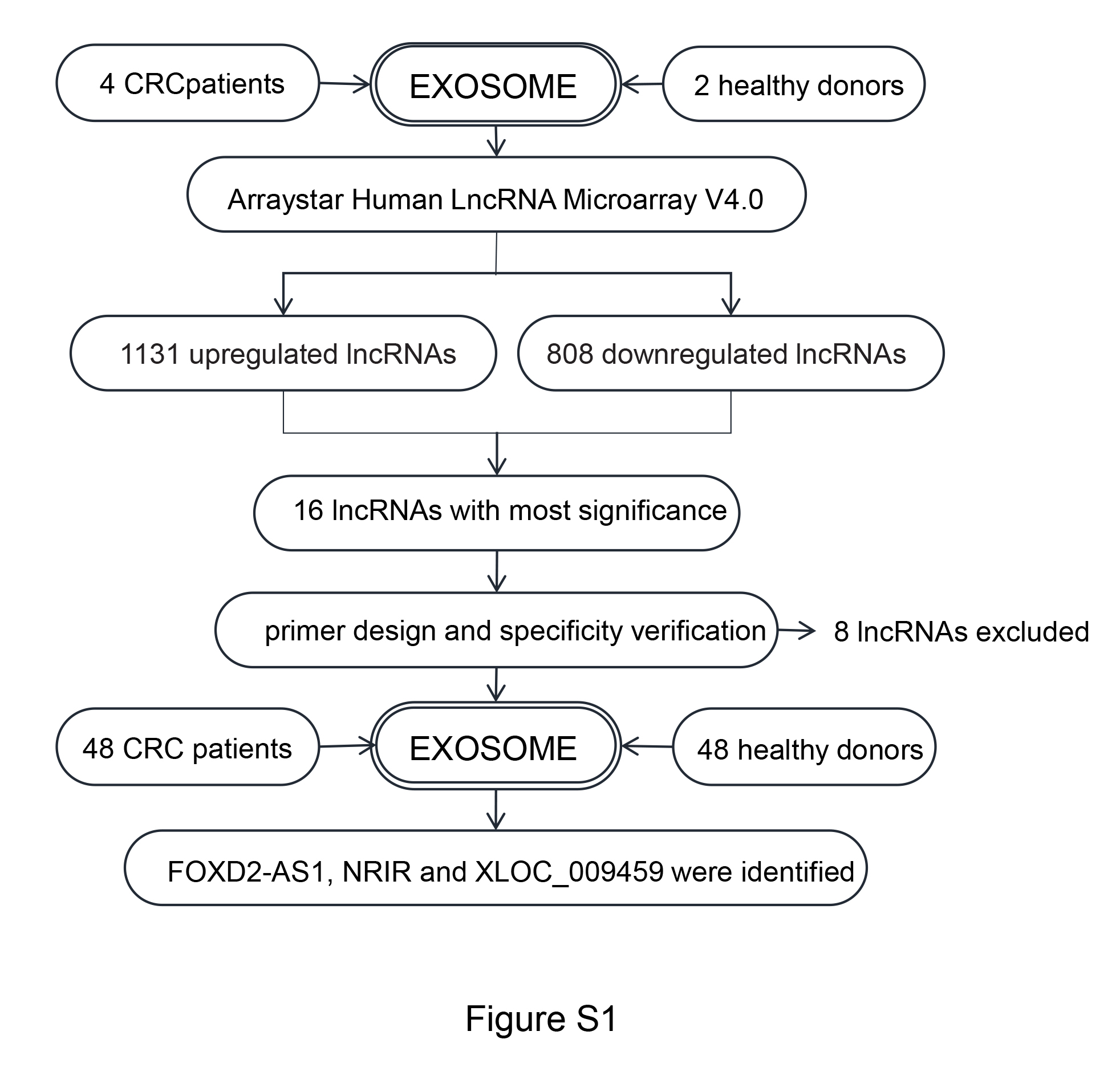

Supplement: Supplementary Figure 1 — The flowchart for the exclusion criteria. [file Image_1.jpeg]

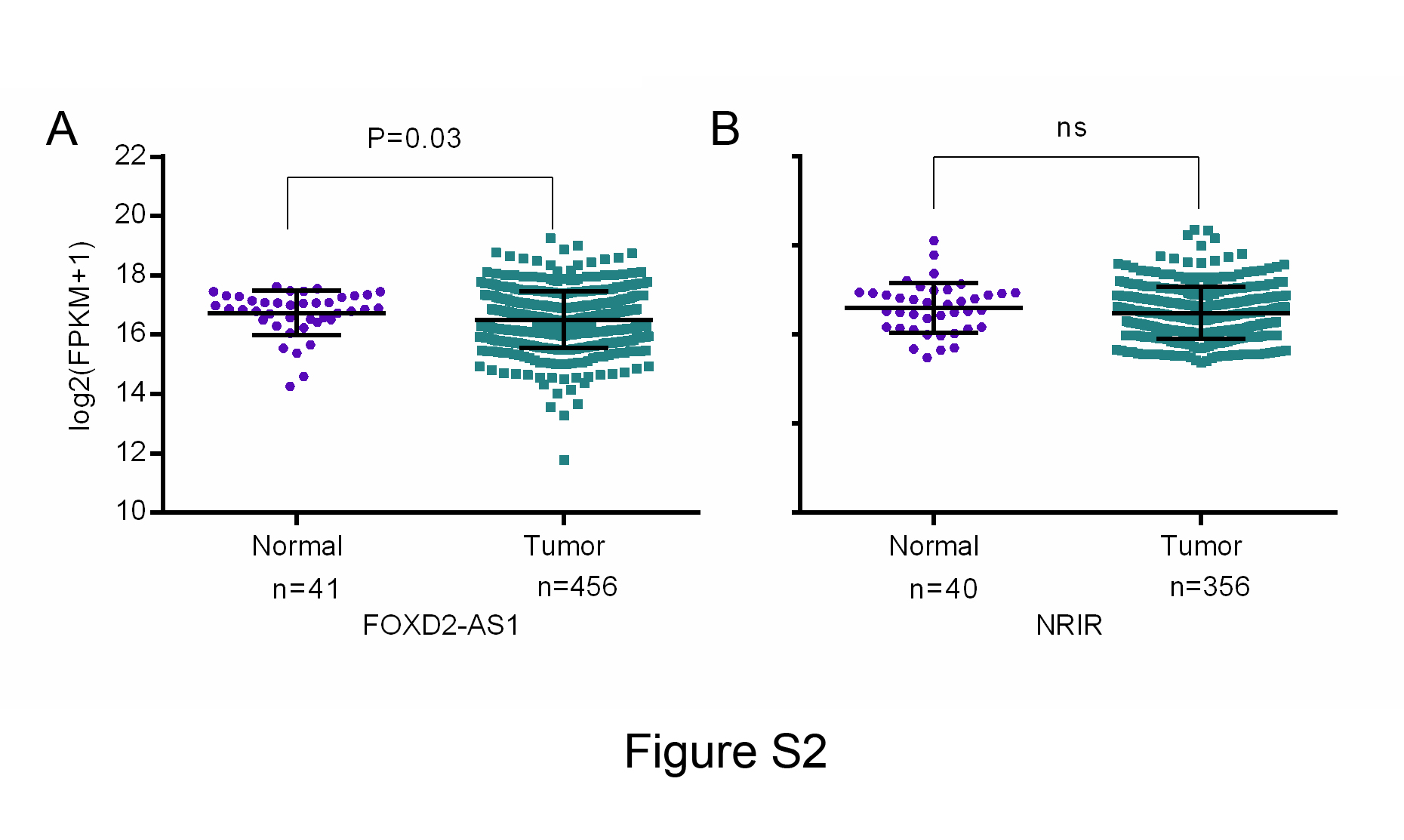

Supplement: Supplementary Figure 2 — The expression of lncRNAs FOXD2-AS1 (A) and NRIR (B) in cancer and paracancerous tissues in the TCGA database. [file Image_2.jpeg]
